# "If you catch my drift...": ability to infer implied meaning is distinct from vocabulary and grammar skills

**DOI:** 10.12688/wellcomeopenres.15210.2

**Published:** 2019-07-10

**Authors:** Alexander C. Wilson, Dorothy V.M. Bishop

**Affiliations:** 1 Department of Experimental Psychology, University of Oxford, Oxford, Oxfordshire, OX2 6GG, UK

**Keywords:** Autism, pragmatic language, social communication, implicature, conversation, psychometric, online research

## Abstract

**Background:** Some individuals with autism find it challenging to use and understand language in conversation, despite having good abilities in core aspects of language such as grammar and vocabulary. This suggests that pragmatic skills (such as understanding implied meanings in conversation) are separable from core language skills. However, it has been surprisingly difficult to demonstrate this dissociation in the general population. We propose that this may be because prior studies have used tasks in which different aspects of language are confounded.

**Methods:** The present study used novel language tasks and factor analysis to test whether pragmatic understanding of implied meaning, as part of a broader domain involving social understanding, is separable from core language skills. 120 adult participants were recruited online to complete a 7-task battery, including a test assessing comprehension of conversational implicature.

**Results:** In confirmatory analysis of a preregistered model, we compared whether the data showed better fit to a two-factor structure (including a “social understanding” and “core language” factor) or a simpler one-factor structure (comprising a general factor). The two-factor model showed significantly better fit.

**Conclusions:** This study supports the view that interpreting context-dependent conversational meaning is partially distinct from core language skills. This has implications for understanding the pragmatic language impairments reported in autism.

## Introduction: Theoretical underpinnings

Observations of people with autism and social communication difficulties suggest that it is possible to have problems with conversational language in the relative absence of impairments in aspects of “core language”, such as vocabulary knowledge and grammatical competence (e.g. Baird & Norbury, 2016; Lam & Yeung, 2012). However, attempts to demonstrate this dissociation using objective tests have mostly failed. Two separate reviews have concluded that conversational skills are closely related to core language abilities ( Andrés-Roqueta & Katsos, 2017; Matthews *et al.*, 2018). However, these findings are based on language tests that may not act as pure indicators of particular language skills. Our goal was to devise novel language tests to give a more convincing answer to the question of whether core language abilities and sensitivity to social aspects of language are separable sets of skills in the general adult population.

While standardised tests of core language skills are in abundance, only a limited range of tests focus on social aspects of language ( Norbury, 2014). Social communication is, of course, a broad construct. It has been defined as the combination of “social interaction, social cognition, pragmatics (verbal and nonverbal) and receptive and expressive language processing” that supports conversation ( Adams, 2005, p. 128). The broad nature of this construct makes it difficult to study with precision. By contrast, *pragmatics* is a specific facet of language processing that can potentially be more easily operationalised for assessment - although, from the offset, we should note that the term has frequently been overextended in the field of communication disorders to essentially mean all social aspects of communication ( Cummings, 2007). In linguistics, pragmatics is defined as “a process of reasoning based on features of context” ( Cummings, 2007, pp. 425–426). The pragmatic aspect of language comprehension mediates between dictionary meaning and a speaker’s communicative meaning). As an example of pragmatics in action, consider the utterance “it’s cold here”. Descriptively, the speaker tells us about the temperature of a place. Through pragmatic processing of the utterance in its particular context, we might also infer that the speaker is implying they want to close a window, for instance, or to go inside. As such, pragmatics involves using context to “read between the lines”.

Existing tests of communication skills are not sufficiently focused to measure pragmatic processing. Communication is often measured globally, through observation of semi-naturalistic conversation (e.g. using the Pragmatic Protocol, Prutting & Kirchner, 1987) or through behavioural checklists completed by informants (e.g. the Children’s Communication Checklist, Bishop, 2003). These assessments are likely to conflate pragmatics, social interaction and core language skills. A further drawback is that they focus on social communication “behaviours” rather than the cognitive functions that underpin them. Pragmatic processing may or may not be responsible for these behaviours. Let’s consider an example: how might we interpret a failure to produce contingent turns in conversation? This was a problem for autistic children assessed using the Yale *in vivo* Pragmatic Protocol, a semi-naturalistic conversational assessment including “specific pragmatic probes” for eliciting target behaviours ( Schoen Simmons *et al.*, 2014). A lack of contingent turns may arise from difficulty inferring what is relevant in the communicative context - i.e. a pragmatic problem. Alternatively, the problem may be a lack of social interest, reduced joint attention, performance anxiety, reduced ideational fluency, problems in finding words, perseveration on one’s own topic of interest, or other issues besides. These are not pragmatic problems.

To quantify pragmatic skills, psychometric assessment is necessary. However, traditional standardised language measures do not pick up pragmatic difficulties ( Conti-Ramsden *et al.*, 1997), and tests designed for this purpose have been quite uninformative. The Test of Pragmatic Language-2 (TOPL-2) is perhaps the most well-known pragmatic test. It requires the individual to produce situationally-appropriate speech acts, and can detect pragmatic impairment in groups with autism ( Young *et al.*, 2005). However, it is not consistently sensitive ( Reichow *et al.*, 2008; Volden & Phillips, 2010). The same problem exists with the pragmatic judgement subtest of the Comprehensive Assessment of Spoken Language, which is a similar, commonly used test ( Klusek *et al.*, 2014). These tests correlate highly with core language skills (e.g. Akbar *et al.*, 2013), and they do not attempt to minimise demands on vocabulary/grammar or expressive language ability, so we should question their specificity as tests of pragmatics. In addition, both these tests focus on politeness and knowledge of social rules, which is different from the linguistic definition of pragmatics, as inference of meaning from context. Another “pragmatic” test, the new Pragma test ( Loukusa *et al.*, 2018), discriminates well between autistic and neurotypical individuals, but is not a pure test of pragmatics, as it contains subscales tapping rather different skills, such as theory-of-mind and emotion recognition. While this kind of test may be useful clinically for picking up communicative problems, it is not well-suited for our purposes, i.e., pinpointing the underlying nature of those problems.

**Figure 1.**
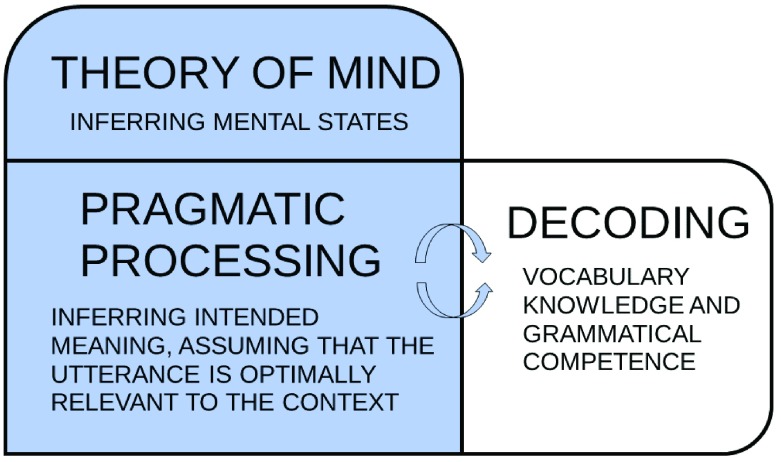
Our model of language processing, as borrowed from Relevance Theory. Language processing is an interaction between decoding and pragmatic inference. This pragmatic processing is seen as a sub-domain of our more general “theory-of-mind” capacity.

We need to be clear what we should measure in a pragmatic test. In reviewing definitions of pragmatics, Ariel (2010) stresses the need to contrast semantics and pragmatics. While semantics involves decoding conventional “dictionary” meaning, pragmatics is all about inference: we use context to infer further non-codified meaning. Please see Figure 1 for a visual representation of our model of language processing. We follow Relevance Theory in assuming that language processing is a combination of (1) decoding the “literal meaning” of an utterance using our vocabulary knowledge and grammatical competence, and – because the linguistic code is always incomplete or ambiguous – (2) inferring from context the full extent of the interlocutor’s intended meaning ( Sperber & Wilson, 1986). In Relevance Theory, inferring communicative meaning is assumed to be a subdomain of our general ability to attribute mental states to others (our “theory-of-mind” or capacity for “mind-reading”). By “subdomain”, we mean that pragmatic processing is thought to happen through a domain-specific heuristic – “communicative relevance” – which, as the name suggests, is specifically activated by communicative behaviour. This means that we infer what an utterance means based on the automatic assumption that interlocutors will communicate in a way that is optimally relevant to the context (see Carston & Powell, 2008).

Relevance Theory assumes that we make inferences at two levels. On the one hand, we need to infer the full “explicit” meaning of particular words and phrases; this is explicature. Partly this is a semantic process, as we need to access the dictionary meaning for the word/phrase; however context is important in determining the appropriate meaning, so this is also a pragmatic process. In addition to explicature, we may also infer further “implicit” meaning, or implicature, from a global understanding of the utterance in context. This involves “reading between the lines” and understanding what has not been directly stated. This is a purer pragmatic process, as it depends wholly on inference. For illustration, consider the following dialogue:

SPEAKER ONE. Can you pick Sally up from the station?
SPEAKER TWO. I’m working all day.

In terms of explicature, “I’m working all day” might be taken to mean “Today I will be doing work tasks at my workplace during my working hours”. Here we make local-level inferences about what the speaker’s individual words mean in context. In addition, we might derive implicature through a global understanding of the utterance in its communicative context. For instance, “I’m working all day” implicitly turns down SPEAKER ONE’s request to pick Sally up. In other contexts, these words would not communicate anything about picking a person up from the station: the implicated, indirect meaning is only expressed here because we assume that an utterance in this context must relevantly address SPEAKER ONE’s question, and so we actively seek an implied meaning.

In designing a theoretically-motivated test of pragmatics, implicature is a good focus, as implicated meaning can only be interpreted through context-dependent inference - i.e. pragmatic processing. Existing research has not explored conversational implicature as discussed above. Generally, empirical research investigating implicature has focused on generalised conversational implicature, especially scalar implicature (where we infer, for instance, that not all the apples are moldy in the sentence “Some of these apples are moldy”). However, with generalised conversational implicature, the implied meaning is invariably present whenever this sort of language is used (e.g. Carston, 1998). Therefore, it is very different from the more particularised conversational implicature discussed above, which is much more dependent on the communicative context.

While research into implicature has generally not been strongly influenced by Relevance Theory, a set of papers by Leinonen, Loukusa and others is an exception ( Leinonen *et al.*, 2003; Loukusa *et al.*, 2007a, Loukusa *et al.*, 2007b; Ryder & Leinonen, 2014; Ryder *et al.*, 2008). These researchers report reduced ability in children with language impairments or autism to make inferences on language tests designed based on concepts from Relevance Theory. However, these studies did not focus on implicature in conversational contexts; they focused on making elaborative inferences to fill in semantic gaps in short story material. This focus is likely to miss the more social aspects of implied meanings, which are important for understanding pragmatic problems affecting everyday communication.

## Introduction: Empirical work

Our objective was to develop a test of pragmatic language processing that removes as much as possible the effect of grammar/vocabulary skills on scores by (a) using simple language and (b) incorporating control items (see below). We focused on implicature comprehension, as linguists are unanimous in viewing implicature as a complex pragmatic phenomenon (e.g. Ariel, 2010). As well as allowing us to test how far pragmatic skill is separable from other language abilities, the test was designed to be clinically applicable in identifying impairments characteristic of autism and social (pragmatic) communication disorder (SPCD) (e.g. Loukusa & Moilanen, 2009), and to be sensitive to developmental and individual differences. Based on these goals, we were mindful of making our Implicature Comprehension Test (ICT) sufficiently child-friendly, while avoiding ceiling effects in adults.

In a pilot study (see Methods), we found that the ICT was internally consistent, and that scores did not correlate significantly with semantic knowledge (measured by tests of vocabulary and recognition of conversational phrases/idioms). This provided some evidence that implicature comprehension dissociates from basic linguistic decoding. In the present study, we set out to collect some more normative data on the ICT in typical adults to replicate that finding, and also to test for a dissociation between implicature comprehension and grammatical ability too. This was our key question: is pragmatic processing (as measured by the ICT) separable from core language skills (grammar and vocabulary)? Our secondary question was whether performance on the ICT was related to performance on other tests measuring social understanding in conversational and other contexts. As noted above, and shown in Figure 1, the capacity to infer intended meanings from context – pragmatic comprehension – is thought to be a subdomain of our more general “theory-of-mind”. Conceptually, then, we would expect our test of pragmatic comprehension – the ICT – to cluster with other tests requiring social inferences in conversational and other contexts, as they involve “theory-of-mind”. As such, we developed a test battery including two tasks that we expected to require sensitivity to conversational contexts as well as a test requiring the individual to infer mental states in different scenarios.

We hypothesised that a two-factor model (“core language competence” and “social understanding”) would account for individuals’ performance across our tests. Reviews of existing research indicates that “pragmatic” measures tend to correlate with tests of core language skills ( Andrés-Roqueta & Katsos, 2017; Matthews *et al.*, 2018), although we should bear in mind that these “pragmatic” measures suffer from problems of poor specificity as noted above, which may inflate the correlations. However, the possibility that individuals can have relatively specific difficulties with pragmatics ( Lam & Yeung, 2012) led us to expect that a two-factor structure would fit the battery better than a one-factor structure where all the tests measured some general ability.

## Methods

This study was preregistered on the Open Science Framework (OSF) with the title “The relationship between comprehension of conversational implicature, core language skills and social cognition” ( Wilson & Bishop, 2018): . The study was granted ethical clearance in July 2018 by the Medical Sciences Interdivisional Research Ethics Committee at the University of Oxford: Ref. R57087/RE002. We report below how we determined our sample size, all data exclusions, all manipulations, and all measures in the study.

### Participants

We recruited 120 adults online via the participant recruitment platform, Prolific, who all gave informed consent to participate. Inclusion criteria included: (i) age over 18, (ii) private access to a computer with a good internet connection, (iii) no significant visual or hearing impairment, (iv) native-level fluency in English, and (v) no participation in the pilot study of this project. Of the 120 participants, 8 were excluded based on poor performance on at least one test; see our exclusion criteria under Data analysis below.

Our sample size was based on a power calculation using simulations. We used a p-value of 0.05 to indicate statistical significance: this reflects a single preregistered statistical test for the confirmatory factor analysis (CFA) used in this study. In determining power for the CFA, we simulated data conforming to a two-factor correlated traits model with a core language factor relating to three tests and a conversation comprehension factor relating to four tests. Factor loadings were set at 0.7 and the factors were correlated at 0.2 (a low correlation was used, as the semantic tests did not correlate with the ICT in the pilot study). In 10,000 simulations of samples of 120 individuals, 9989 datasets showed a significantly better fit to a two-factor compared to a one-factor model, when compared using a chi-square test.

In an open response format, we asked participants to give their age, gender and race/ethnicity. Mean age of the participants was 30 years; 11 months (SD = 11 years; 3 months, range = 18 – 64 years). 65 identified as women, 54 as men, and 1 person did not declare their gender. The majority of the sample described themselves as White (103 out of 120); 4 people identified as Mixed Race, 4 as Black, and 8 as Asian. We also asked participants whether they were currently studying. 34 of the participants said they were, of whom 28 reported they were completing undergraduate studies and 6 indicated they were postgraduates. Of the 86 individuals who reported not being students, highest level of education was given as high school/secondary school for 18 individuals, vocational training/college courses for 13, bachelor’s degree for 53, and a higher degree for 9.

### Procedure

This was an online study supported by Gorilla and all data were collected via Prolific on 2 ^nd^ August 2018. Participants completed the study at a time and place of their convenience. The study took around 30–45 minutes to complete and individuals were remunerated £5 for their time.

### Measures

Seven short language tasks were presented in the order described below. See Extended data ( Wilson & Bishop, 2019) for examples of test items.

**Implicature Comprehension** was measured using the Implicature Comprehension Test (ICT). The task involves watching a series of 57 short cartoon videos (each is approximately 8 s in length). Each video consists of a conversational adjacency pair between two characters: typically this is a closed question from Character 1 (eliciting a “yes” or “no” response) followed by a response from Character 2. Each utterance is between 6 and 8 words in length, and age of acquisition of the words does not exceed middle primary school level. Following the adjacency pair, the participant hears a comprehension question; typically, this echoes the question posed by Character 1 during the video. The participant then hears a bleep, and is asked to give a “yes”-“no”-“don’t know” response to the question, recorded by keyboard presses. There is a 5-second limit for responding. Note that there were few instances of time-outs in this study. Among the 117 participants whose data were included for this task, responses were missing for only 0.5% of trials. The most trials missed by any one participant was 4, and 94% of participants missed one or fewer trials (83% did not miss any). Five example items (one of the practice trials and four implicature items) are available as Extended data ( Wilson & Bishop, 2019).

In 27 or the 57 trials, Character 2’s response provides an answer to Character 1’s question that must be inferred via implicature. Half of these represent a “yes” response; half a “no” response.

Example: Character 1: “Could you hear what the police said?” Character 2: “There were lots of trains going past.” Comprehension Question: “Do you think she heard what the police said?” Correct Answer: “No.”

In a further 10 trials, Character 2’s response provides a more explicit response to Character 1’s question. The structure of these items and the overall language level was similar to the implicature items, and therefore they represent positive control items, designed to check that basic language comprehension and task structure did not cause any problems. In this study, mean accuracy on these items was 95.4% (SD= 9.95%), indicating that these items functioned well.

Example: Character 1: “Did you see the policemen earlier on?” Character 2: “I saw them standing on the platform.” Comprehension Question: “Do you think he saw the policemen?” Correct Answer: “Yes.”

In a further 10 trials, Character 2 provides a “don’t know” response to the question, and for these trials, the correct response to the follow-up comprehension question is “don’t know”. These trials are designed to legitimize “don’t know” responding so that participants don’t feel that providing a “don’t know” response always represents an incorrect answer. The aim of this is that participants who are less sensitive to implicature are likely to provide “don’t know” responses to implicature items too. These trials functioned well in getting participants to use the “don’t know” response; mean accuracy on these items was 87.5% (SD= 20.1%).

Example: Character 1: “Did the police speak to anyone else?” Character 2: “I wasn’t watching them much.” Comprehension Question: “Do you think the police spoke to anyone else?” Correct Answer: “Don’t know.”

Finally, the task involves a further 10 “open context” implicature items. In these items, Character 1 produces a statement rather than a question. Character 2’s response implicitly addresses Character 1’s statement. The follow-up comprehension question assesses whether participants have appreciated the implicature; half of the comprehension questions are correctly answered by “yes”, half by “no”.

Example: Character 1: “Normally the station doesn’t get busy.” Character 2: “Lots of people were coming on holiday today.” Comprehension Question: “Do you think the station was busy?” Correct Answer: “Yes.”

Utterance length and psycholinguistic variables (word frequency, word age-of-acquisition and word concreteness) are controlled for the different item types. Mental state words are also avoided, to remove the “theory-of-mind” demand of these. There were two measured variables: the sum of implicature items correctly answered (out of 37) and the sum of control items correctly answered (out of 10).

**Receptive vocabulary** was measured by a Synonyms Test devised for this study. This includes 25 items. In each trial, participants choose which of four words is a synonym for the target word. This is a timed task (up to 12 s per item). There was one measured variable: the sum of items correctly answered (out of 25).

**Receptive grammar** was measured by a Grammaticality Decision Test devised for this study. In this task, participants listen to sentences and decide if they are well-formed and grammatical. There are 50 items and half are grammatical. Grammatical violations represent mistakes that native speakers would not tend to make, such as using an incorrect auxiliary verbs (e.g. “If I will see Ann today, I’ll ask her opinion) or atypical placing of adverbs (e.g.”If you can’t find it, I can send again the letter“). Participants have up to 6 seconds to listen to each sentence and indicate by a button press whether or not it is grammatical. There was one measured variable: the sum of items currently answered (out of 50).

**Sensitivity to social awkwardness** was measured using the Awkward Dialogues. This task is based loosely on the Faux Pas Recognition Test ( Baron-Cohen *et al*., 1999). Individuals needed to detect discomfort or offence implicitly conveyed in short dialogues. The test was designed not so much to measure a single skill but rather to tap general conversational competence, including pragmatics, mental state attribution, and understanding of paralinguistic cues such as intonation, as well as core language skills.

In the Awkward Dialogues, participants listen to eight short dialogues of around 80–90 words in which two characters each take five conversational turns. In five of the dialogues, one of the characters says something that is socially awkward. Three of the dialogues are control stimuli, in which nothing awkward is said. Participants need to indicate whether something awkward was said, and if they indicate that it was, they are asked to produce a written response to explain what was awkward, why the interlocutors spoke as they did and how they might have felt.

Participant responses on the five awkward dialogues were marked out of two by the first author. Two marks were given if the response expressed the main point that made the dialogue awkward, one if the response gave a glimmer of a correct answer, and zero if the participant missed the awkwardness in the dialogue. Responses from 50 participants were checked by a second independent marker; Cohen’s Kappa was 0.78 indicating good inter-rater reliability. Scores for the five awkward dialogues were entered into an item response model, so that factor scores could be extracted as our measure of participant ability on this test. We chose to use factor scores rather than sum totals, since it was not clear that the intervals in the mark-scheme (i.e. between 0, 1 and 2) ought to be seen as equal. Using a polytomous item response model allowed a solution to this issue, as such models treat data as ordinal and model participant ability as a function of the pattern of responses across items.

In addition to being asked whether dialogues are socially awkward, participants are asked a factual recall question to check basic comprehension of each dialogue (1 mark for each of 5 questions). Participants were excluded if they incorrectly answered more than one factual recall question, as these individuals were outliers (according to the outlier definition below). Where participants incorrectly answered one factual recall question and did not score two marks for that dialogue, their mark for that item was deleted and re-imputed by the item response model based on their scores for the other four awkward dialogues.

**Comprehension of fillers/backchannel continuers** was measured using a Test of Fillers and Backchannels devised for this study. We expected this test to require sensitivity to the role of conversational fillers in turn-taking. Fillers are thought to have functions in conversation; they are not merely “rubbish” produced by inefficient language production systems. For instance, “um” and “uh” are used to claim or hold the floor ( Clark & Fox Tree, 2002). Meanwhile, backchannel continuers, such as “mhmm” and “uhuh”, cede the floor to the interlocutor ( Jurafsky *et al.*, 1998). As these examples indicate, many fillers are non-lexical speech sounds which do not have substantive meaning but which nonetheless have a communicative function in negotiating who speaks in an interchange. We expected comprehension of fillers/backchannels to allow quite a “pure” test of sensitivity to the turn-taking mechanics of conversation, as this should make minimal demand on core language skills (i.e. vocabulary and grammar), especially given the non-lexical nature of many fillers/continuers.

Participants watch short videos in which Character 1 makes an utterance of between 5 and 9 words. Next, Character 2 produces a word or non-lexical speech sound. The video then cuts off before anything else can be heard. Participants need to indicate who they think would speak immediately after what is observed in the video. They provide their answer by clicking a button showing the face of the character. A third button shows a question mark that participants may click if it is “very difficult to say”. The task includes 40 items. Character 1 says 20 different utterances in the course of the task, with each one produced twice. Character 2 follows up the utterance with a backchannel continuer (or the repair initiator “huh?”) one time and a filler claiming the floor the other time. Where a backchannel continuer or “huh?” is used, participants need to select Character 1 as the person likely to speak, and Character 2 where a filler claiming the floor is used. There are 4 backchannel continuers (mm-hmm, uh-huh, really, right) and 5 fillers claiming the floor (um, uh, yeah, oh, well). The backchannel continuers are much more often used in this role than to signal an incipient speaker (e.g. in corpus analysis by Jurafsky *et al.*, 1998). With the five fillers, “um” and “uh” are taken to mark a slight pause in which Character 2 formulates what they want to say, and “yeah”, “oh” and “well” are fillers claiming the floor. “Yeah” can be used as a backchannel in conversation, though it is much more commonly used to claim the floor than other backchannels ( Drummond & Hopper, 1993) and its function is often signaled by the intensity with which it is spoken ( Trouvain & Truong, 2012). We therefore use prosodic information to disambiguate “yeah” (we do the same for “oh”, since this this is likely to function similarly to “yeah”). There was one measured variable: the sum of items correctly answered (out of 40).

**Narrative-based inferencing** was measured by a Test of Local Textual Inference devised for this study. In this study, we were interested in narrative-based inferencing, as the relationship between this kind of inferencing and other language skills was of theoretical interest. In processing narrative (or spoken/written discourse more generally), it is assumed that we construct a coherent mental representation of a narrative based on its explicit content, while making inferences to fill any gaps using text-based cues and world knowledge (e.g. Garnham & Oakhill, 1992). This type of inferencing depends heavily on core language skills, such as semantics ( Adams *et al.*, 2009; Bishop & Adams, 1992; Botting & Adams, 2005; Lucas & Norbury, 2014). We expected narrative-based inferencing to be quite a different process to interpreting an implicated meaning in a two-way conversational context. For the latter, a key feature is that we need to understand what is expected of a speaker such that their turn is relevant at the particular point in the conversation. As such, we expected to find a dissociation between narrative-based inferencing and comprehension of conversational implicature. Since Relevance Theory stipulates that all language is underdetermined and requires inferences to be made at the local level to enrich and disambiguate the utterance, we expected this type of inferencing to reflect core language competence.

In this task, participants read two 100-word sections of a short story and after each section they respond to ten questions with a word or short phrase. Participants have as long as they like to read the text, and then up to 25 seconds to type their response to each individually-presented question. The text remains on the screen when the questions are asked. The questions assess whether participants can make coherence inferences to build up a comprehensive representation of a text. Participants are informed that they may respond that they don’t know the response to a question (if there’s no relevant information, for example). Four questions are correctly answered by “don’t know”. Two marks were awarded for a correct response and one mark for a partially correct response, making a maximum total of 40 points.

**Mental state attribution** was measured using the Frith-Happé Animations ( Abell *et al.*, 2000). Each animation shows two moving triangles that sometimes interact. In this study, individuals were presented with a shorter version of the task, which included all the animations showing a “theory-of-mind” scenario or goal-directed behaviour; we did not use those animations depicting random movement. The “theory-of-mind” animations show the triangles interacting as if they are trying to influence the thoughts and feelings of each other. The “goal-directed” animations show the triangles physically interacting (e.g. fighting). In this version of the task, participants watched animations around 20 s in length (original clips were shortened), before providing a typed answer describing what happened in each animation. As in the original instructions, participants were told that the triangles would interact, and sometimes they would interact as if they were aware of each other’s thoughts and feelings. There were five “theory-of-mind” trials (the four in the original task, plus one of the items originally designated as practice) and four goal-directed trials. We gave the original goal-directed practice item as our single practice item. Each of the participant’s written descriptions were scored on their appropriateness (i.e. how accurately they inferred the scenario the cartoon represents) out of 3 by the first author according to the mark scheme used by Castelli *et al.* (2000). A second independent marker also scored the responses given by 50 participants; Cohen’s Kappa was 0.79 indicating good inter-rater reliability. Scores for the “theory-of-mind” animations were entered into an item response model, for the same reason as the scores for the Awkward Dialogues, and factor scores were used as our measured variable for this task.

### Pilot study

Prior to our main study, we wanted to establish the reliability of our Implicature Comprehension Test and the distribution of scores expected in the general adult population. We also planned to assess how implicature processing relates to other language skills and to the broad autism phenotype.

We conducted an online study supported by Gorilla, using Prolific as a platform for recruiting participants in May 2018. We recruited 120 adults who reported speaking English as a first language and living in the UK. Individuals were excluded if they reported a significant uncorrected hearing or visual impairment. For our preregistered protocol, please see https://osf.io/t54hm/. The study was granted ethics clearance by the Medical Science Interdivisional Research Ethics Committee at Oxford University in April 2018 (Ref: R57087/RE001). Participants completed four language tests: the Implicature Comprehension Test (ICT) and Synonyms Test, which were also administered in the main study, and also tests of syntax and idiom recognition (see below). In the pilot study, there was no time limit for responses on the ICT. Participants also completed the Autism-Spectrum Quotient (AQ), a self-report measure of autistic traits ( Baron-Cohen *et al.*, 2001).

**Receptive syntax processing** was measured by the Test of Complex Syntax-Electronic (TESC-E; Frizelle *et al*, 2017; see https://osf.io/5ntvc/ for full details). Participants watch a series of videos and for each video decide whether an auditorily-presented sentence correctly describes activity in it. The task was originally designed for use with children, and so we incorporated some adaptations to make it more appropriate for adults. In the original version, accuracy was the variable of interest, though we expected a ceiling effect for accuracy, so we instead focused on mean reaction time. In making this change, we needed to present the sentence at the end of the video rather than during it, which was the original format; this meant we could time an individual’s response from the onset of the sentence. Furthermore, we only included the relative clause items, and not the adverbial clause or sentential complement ones. The adverbial clause items make too high a demand on memory when the video and audio are not presented simultaneously, and the sentential complement items (e.g. “She thinks [that] …”, “He wishes [that] …”) are likely to demand “theory-of-mind” processing that ideally should be removed from this task to ensure that it is a relatively pure measure of structural language skills. In this adapted form of the task, participants watch a series of 20 videos, each around 4 seconds (shorter than in the original version to increase the speed of the task for adults). After each video, participants hear a biclausal sentence incorporating a main clause and a relative clause. There are five types of relative clauses: transitive subject relatives, intransitive subject relatives, direct object relatives, indirect object relatives, and oblique relatives. Participants indicate via keyboard presses whether the sentence correctly describes the video.

**Idiom recognition** was measured by an Idiom Decision Test devised for this study. Participants are presented with 40 three-word phrases, each including a transitive verb and its direct object. Half the phrases are idioms (i.e. conventionalised phrases with a meaning that extends beyond the meaning of the component words; e.g. “make your day”) and half are incorrect adaptations of common idioms (e.g. “bite the dirt”, instead of “bite the dust”). Idioms are presented via audio, and participants need to decide as quickly as they can if the phrase is an idiom or not. Responses are given by keyboard presses. Our variable of interest was total accuracy (1 point per correct answer).

See Table 1 for descriptive statistics and the reliability of our measures.

**Table 1.** Descriptive statistics, excluded values, and reliability coefficients (Cronbach’s alpha and standard error of measurement). ICT - Implicature Comprehension Test, RT – response time.

	N	Mean	SD	Min	Max	Skew	Kurtosis	Exclusions	Alpha	SEm
ICT, Accuracy on explicit items	118	9	0.63	8	10	-1.38	0.72	6, 1		
ICT, Accuracy on implicatures	118	27.2	4.08	14	35	-0.79	0.37	29, 2	0.80	1.82
Accuracy on synonyms	120	13.4	5.34	3	25	0.12	-1.03		0.85	2.07
Accuracy on idioms	113	35.8	3.19	26	40	-1.31	1.23	16, 18, 24, 23, 22, 20, 17	0.83	1.32
Accuracy on syntax	114	19.3	0.80	17	20	-1.01	0.46	16, 20, 17, 16, 13, 11		
Mean RT on syntax (ms)	114	3320	447	2188	4721	0.77	0.81	2780, 5375, 6131, 4334, 3572, 1231	0.84	178.64
Total score (out of 50) on AQ	118	20.7	6.92	5	39	0.33	-0.23	NA, NA	0.79	3.17

For each variable, we excluded scores according to the criteria of Hoaglin & Inglewicz (1987) for outlier exclusion: 2.2 times the interquartile range below the lower quartile (and above the upper quartile in the case of syntax RT). In analysing the data, we used listwise deletion of any cases with an outlying score. See Table 2 for Spearman’s correlations between the five test variables.

**Table 2.** Correlations between language tests and Autism-Spectrum Quotient (AQ). There has been listwise deletion of cases with outlying scores (N = 108). RT – response time.

	Accuracy on synonyms	Accuracy on idioms	Mean RT on syntax	Total score on AQ
Accuracy on implicatures	-0.04	-0.10	0.01	-0.09
Accuracy on synonyms		0.49	0.25	0.04
Accuracy on idioms			0.24	0.12
Mean RT on syntax				0.02

The correlation matrix suggests that the synonym and idiom tests measured closely related semantic skills, and that the syntax test was loosely related to these tests. It is worth noting that the syntax task seemed to have been overly simple for typical adults (as indicated by the strong ceiling effect on accuracy), and so the main demand is unlikely to have been language processing – this perhaps accounts for the low correlations observed between it and the other language tests. Implicature scores on the ICT were not well-correlated with scores on any other tests. We cannot attribute this to noisy tests, as reliability was consistently good across the tests, as indicated by the Cronbach’s alpha values. Similarly, there was no issue with restricted variance on the ICT; while scores were a bit skewed towards the maximum mark, there was nonetheless substantial variability. Clearly the skills assessed by the ICT were not closely related to other skills measured by the test battery. Likewise, the AQ was not closely related to the other tests, suggesting that the broad autism phenotype was not associated with language skills tested here. This was unexpected, as we thought AQ scores would be negatively related to ICT scores, as autism has been related to difficulties processing implied and indirect meanings. As noted in the main body of the paper, the lack of correlations between the ICT and other tests left us unsure what the test was measuring: very task-specific skills or a more general competence in conversational understanding? This question motivated the follow-up work reported in the main study.

### Data analysis

Data were analysed using R version 3.4.0 ( R Core Team, 2017); We used the following R packages as part of our data analysis: psych version 1.7.8 ( Revelle, 2017), mirt version 1.28 ( Chalmers, 2012), MVN version 5.5 ( Korkmaz *et al.*, 2014), rcompanion version 1.13.2 ( Mangiafico, 2018), and lavaan version 0.6.4.1348 ( Rosseel, 2012). Plots were produced using the following packages: ggplot2 version 2.2.1 ( Wickham, 2009) and SemPlot version 1.1 ( Epskamp & Stuber, 2017). knitr version 1.21 ( Xie, 2017), papaja version 0.1.0.9842 ( Aust & Barth, 2018), weights version 0.85 ( Pasek, 2016), and htmlTable version 1.11.2 ( Gordon *et al.*, 2018) were used to produce the Rmarkdown report for this project. Our data are accessible on OSF (Underlying data ( Wilson & Bishop, 2019)).

**Figure 2.**
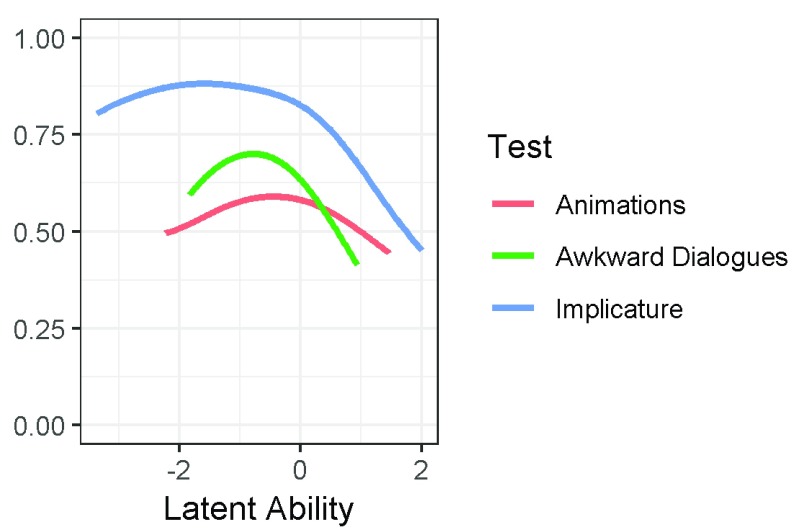
Reliability curves based on item-response theory (IRT) modelling. These curves show test reliability across the ability spectrum for implicature comprehension, sensitivity to social awkwardness and mental state attribution.

We assessed item functioning and the reliability of our measures using classical test theory (CTT) and item response theory (IRT; Embretson & Reise, 2000). The purpose of this analysis was to reduce the amount of measurement error in our tests, so that scores reflected as far as possible participants’ “true scores” in the particular domain being measured. This was particularly important as our tests were novel, and not standardised measures with established psychometric properties. We therefore needed to establish that they were good quality measures. (Note that while there are standardised measures of vocabulary and grammar available, these generally can’t be replicated in an online format due to copyright – and indeed, are not validated for online use.) For each item in each test, we inspected accuracy and the correlation between item-accuracy and total-accuracy on the test with that item excluded (all item-total correlations calculated in this study used test totals with the item excluded). Items were identified as poor if they had low accuracy and a low item-total correlation. We also inspected item characteristic curves (ICCs) produced using IRT analysis, which determines difficulty and discrimination parameters for each item. ICCs are useful in identifying poor items, as they show the probability that an individual of a certain ability scores at a particular level on an item; low flat curves indicate items that are ambiguous, with no consensus answer at any point along the ability spectrum. We excluded any items showing this pattern. We then computed the reliability of our measures using CTT coefficients (Cronbach’s alpha and standard error of measurement). We report these below, alongside a summary of the item-total correlations and item-level accuracy for each test; we summarise these as median and upper and lower quartiles. Please note that this CTT reliability analysis was not done for the Awkward Dialogues or Frith-Happé Animations, as we derived factor scores from IRT models for these tests, and therefore used IRT reliability analysis (explained with Figure 2 below).

Any individual with at least one outlying score on any test was excluded from the dataset. Outliers were defined according to Hoaglin & Iglewicz (1987) as 2.2 times the interquartile range below the lower quartile. We also excluded any individuals who had outlying scores on the explicit-response items of the ICT (this threshold was 8/10) or on the factual questions of the Awkward Dialogues Task (4/5), as these were taken to be control variables identifying individuals who did not engage well with the tasks. Then we inspected the data for univariate normality and multivariate outliers. Multivariate outliers were defined as individuals whose adjusted Mahalanobis’ distance was above the 97.5th percentile of the chi-distribution. Where necessary, we transformed variables using the Tukey ladder of power transformations to reduce skew.

We had one preregistered analysis for this study: a confirmatory factor analysis (CFA). We specified a two-factor correlated-traits model with a “core language” factor and a “social understanding” factor; see Table 3 for which variables were set to load on which factor. We used maximum likelihood estimation with robust standard errors and a Satorra-Bentler corrected chi-square test to evaluate whether a two-factor model fitted the data significantly better than a one-factor model on which all seven variables were set to load on a single factor. The conventional alpha level of 0.05 was used to indicate statistical significance, as we only preregistered one statistical test. We report confirmatory fit indices (CFIs) and root mean square error of estimation (RMSEA) with 90% confidence intervals.

**Table 3.** Study variables for each factor.

Test	Study Variable	Details
**Social Understanding**		
Implicature Comprehension Test	Implicature Comprehension	Total score (36 items, 1 point each)
Test of Fillers/Backchannels	Comprehension of Fillers/Backchannels	Total score (40 items, 1 point each)
Awkward Dialogues	Sensitivity to Social Awkwardness	Factor score derived from IRT model
FrithHappé Animations	Mental State Attribution	Factor score derived from IRT model
**Core Language**		
Test of Local-level Inferencing	Narrative-based Inferencing	Total score (20 items, 2 points each)
Grammaticality Decision Test	Receptive Grammar	Total score (44 items, 1 point each)
Synonyms Test	Receptive Vocabulary	Total score (25 items, 1 point each)

We carried out one exploratory analysis. This was intended as a test of the extent to which implicature comprehension overlapped with core language skills (with which we expected minimal overlap) and other tests involving social understanding/inferential skills (with which we expected more overlap). Essentially, we were looking at whether the ability measured by the implicature comprehension test was more specific or more general, as a means of understanding more about the construct we were measuring. As such, we quantified the proportion of variance in implicature scores predicted by our tests tapping social understanding/inferential skills over and above the effect of core language skills. We ran a hierarchical multiple regression, entering receptive vocabulary and receptive grammar as predictors of implicature comprehension in the first stage. In the second stage, comprehension of fillers/backchannels, sensitivity to social awkwardness, narrative-based inferencing, and mental state attribution were added to the model. We report F-statistics, p-values, and adjusted R-square values for the stages.

## Results

Table 4 shows descriptive statistics for each language measure. The scores for the Frith-Happé Animations and the Awkward Dialogues are IRT factor scores; for context, the mean raw total for the former (just the “theory-of-mind” animations) was 10.04 out of 15 (SD= 2.59) and for the Awkward Dialogues was 6.61 out of 10 (SD= 2.51).

**Table 4.** Descriptive Statistics. We report all excluded values for each study variable. (One excluded value for Sensitivity to Social Awkwardness is NA, as the participant provided no responses to any dialogue.)

	N	Mean	SD	Minimum	Maximum Achieved	Maximum Possible	Skew	Kurtosis	Exclusions
Implicature	117	28.9	4.50	8	36	36	-1.20	2.79	13 20 27
Fillers/Backchannels	120	26.8	5.93	7	37	40	-0.83	0.79	
Social Awkwardness	118	0.01	0.75	-1.83	0.94	0.94	-0.57	-0.57	-1.1 NA
Mental State Attribution	120	0.00	0.74	-2.23	1.47	1.47	-0.38	-0.18	
Inferencing	120	34.4	3.24	24	40	40	-0.73	0.30	3 12 19 19 20
Grammar	118	35.2	4.21	25	44	44	-0.52	-0.37	15 19
Vocabulary	120	12.1	4.45	3	24	25	0.31	-0.26	

### Reliability analysis

When assessing item functioning, we found a few weak items in the grammaticality test: six showed chance-level accuracy and low item-total correlations, and their ICCs were low and flat. This suggests that the six items were ambiguous, with judgements of grammaticality being essentially random across the ability range, so they were dropped from analysis; maximum score on the final version of the test was therefore 44. In the ICT, there was one similarly poor item, and it was removed from analysis. Maximum score on this test became 36. We did not detect any issues with item functioning in any other test.

The final versions of the tests were reliable, as indicated by the CTT reliability coefficients shown in Table 5. It is notable that item-level accuracy was quite variable on the tests. For some items, there was very high agreement between participants; these items were clearly very easy to interpret or involved highly salient communicative cues. Other items were more difficult. These more difficult items correlated well with total scores excluding that item, indicating that they reliably tapped a particular skill.

**Table 5.** Reliability Analysis, including Cronbach’s alpha, standard error of measurement (SEm), corrected item-total correlations (totals excluding the item), and item-level accuracy.

			Item-total correlations			Item-level Accuracy		
Study Variable	Alpha	SEm	Lower quartile	Median	Upper quartile	Lower quartile	Median	Upper quartile
Implicature	0.80	2.01	0.23	0.30	0.38	0.68	0.86	0.91
Fillers/Backchannels	0.80	2.65	0.2	0.27	0.35	0.51	0.67	0.85
Inferencing	0.75	2.63	0.36	0.27	0.36	1.6	1.76	1.86
Grammar	0.79	1.93	0.15	0.27	0.36	0.76	0.89	0.93
Vocabulary	0.76	2.18	0.22	0.31	0.35	0.28	0.49	0.58

See Figure 2 for reliability plots of the tests that we analysed using IRT modeling. IRT models compute standard error of measurement as a function of participant ability, and so we can use this to estimate reliability [SEm = SD*sqrt(1-reliability)]. We present reliability curves for the Awkward Dialogues and Frith-Happé Animations, and also include the IRT curve for the ICT, as we were particularly interested in how this task functioned across the ability spectrum. The low number of items (only five) seems to have limited the reliability of the Awkward Dialogues and Frith-Happé Animations. This IRT analysis is only valid insofar as a unidimensional model fits the data, so we report fit indices for these models: Awkward Dialogues CFI= 1.00, RMSEA= 0.06; Frith-Happé Animations CFI= 0.84, RMSEA= 0.00; ICT CFI = 1.00, RMSEA= 0.01.

### Confirmatory factor analyses

As shown in Table 2, a few scores were identified as outliers. We performed listwise deletion of cases including any outliers, meaning that data from 112 individuals were used in the CFAs reported below.

Accuracy on six of the language tests was not normally distributed; the Shapiro-Wilk test was only non-significant for vocabulary. Therefore, the six variables were transformed. Please note that the CFAs reported below were also run using the non-transformed data, and results were very similar. We decided to transform the data because several multivariate outliers were identified in the non-transformed data. Following transformation, there were no multivariate outliers.

Table 6 presents the correlation matrix for the transformed language variables.

**Table 6.** Correlations between Study Variables. Variables have been transformed and there has been listwise deletion of cases to show only the data to be modelled in the Confirmatory Factor Analysis (N = 112).

	Fillers/Backchannels	Social Awkwardness	Mental State Attribution	Inferencing	Grammar	Vocabulary
Implicature	0.38	0.26	0.27	0.22	0.32	0.20
Fillers/Backchannels		0.11	0.19	0.16	0.27	0.11
Social Awkwardness			0.10	0.27	0.22	0.13
Mental State Attribution				0.20	0.09	0.20
Inferencing					0.13	0.13
Grammar						0.46

We ran two CFAs comparing one and two factor models. A two-factor model fitted the data well (CFI = 0.95, RMSEA [90% CIs] = 0.05 [0, 0.11]), whereas a one-factor model did not (CFI = 0.87, RMSEA [90% CIs] = 0.07 [0, 0.13]). The Satorra-Bentler scaled chi-square difference test showed that the two-factor model gave significantly better fit, *χ* ^2^ (1) = 5.48, *p* = 0.02.

**Figure 3.**
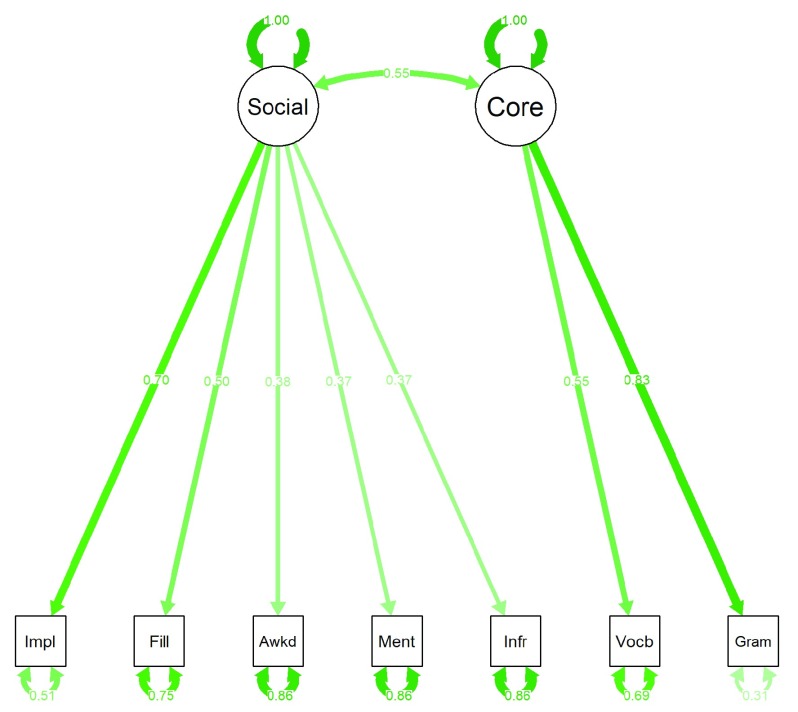
Final two-factor model, with one factor representing social understanding/inferencing and the other factor representing core language skills.

Inspection of the residuals of the two-factor model indicated that there was some mis-specification in this preregistered model. The four highest residuals were all for correlations between inferencing and the pragmatic/social communication tests, indicating that these relationships were not well accounted for by the model. Therefore, we re-specified the two-factor model with vocabulary and grammar loading on one-factor and all the other tests loading on a second factor. This new two-factor model showed good fit (CFI = 1.00, RMSEA [90% CIs] = 0 [0, 0.08]). The Satorra-Bentler scaled chi-square difference test indicated that this two-factor model was significantly better than the one-factor model, *χ* ^2^ (1) = 12.47, *p* < 0.001. See Figure 3 for a visualisation of this two-factor model.

### Exploratory analysis

In addition to our preregistered analysis, we ran a hierarchical multiple regression to explore predictors of implicature comprehension. In the first stage, the full model was significant, F(2, 109) = 6.41, *p* = 0.002. The adjusted R-square value indicated that receptive vocabulary and receptive grammar explained 8.89% of the variance in implicature comprehension. The addition of the rest of the predictors in stage 2 significantly increased the amount of variance explained, F(4, 105) = 5.60, *p* < 0.001, and the full model remained significant, F(6, 105) = 6.23, *p* < 0.001. However, together the variables only explained 22.04% of the variance in implicature comprehension. See Table 7 for the significance of individual predictors. Note that assumptions of multiple regression were checked: residuals were normally distributed and homoscedastic, and there were no influential observations (maximum Cook’s distance = 0.07).

**Table 7.** Coefficients for Multiple Regression, with Implicature Comprehension scores as the criterion variable.

	Estimate	Standard Error	t-value	p-value
**Stage 1**				
Vocabulary	0.03	0.06	0.60	0.553
Grammar	0.14	0.05	2.86	0.005
**Stage 2**				
Vocabulary	0.01	0.06	0.21	0.835
Grammar	0.09	0.05	1.83	0.070
Inferencing	0.22	0.26	0.87	0.387
Social Awkwardness	0.04	0.02	1.64	0.104
Filler/Backchannels	0.04	0.01	3.03	0.003
Mental State Attribution	0.06	0.03	1.89	0.061

## Discussion

Our key preregistered research question was whether the processing of implied meaning (or implicature) in conversation was distinct from core language abilities (i.e. their grammar and vocabulary skills). The low correlations between our implicature comprehension test (ICT) and the grammar and vocabulary tests support the hypothesis that core language skills and pragmatic processing of implicature are separable domains. As such, having well-developed core language skills does not necessarily mean that an individual will be adept at processing conversational implicature. We also set out a preregistered hypothesis that implicature comprehension and other aspects of social understanding would cluster together as a “social understanding” factor, distinct from a core language factor. This hypothesis was supported too, as our data collected in the general adult population showed better fit to a two-factor model than a one-factor model. This means that rather than all the tests reflecting a single general factor, we found evidence in the pattern of correlations that core language skills (grammar and vocabulary) and social understanding (including implicature comprehension) represented partially separable domains. While this was the case, the tests clustering under the social understanding factor showed only relatively low correlations with each other. Therefore, it would be most accurate to speak of these tests as only partly reflecting a general ability, with skills specific to the individual tests being most influential in determining how well people performed on them.

Although our preregistered hypotheses were supported, it should be noted that model fit was improved when one of the three tests set to load on the "core language" factor (narrative-based inferencing) was modeled as part of the other factor instead. This does raise questions about what this factor represents. The first three tests loading on this factor required individuals to interpret how conversational partners communicate implicitly, how they use fillers to negotiate conversational turns, and how they convey social discomfort/offence to each other. These tests all required sensitivity to a speakers’ communicative intents in conversational contexts. In the fourth test, individuals needed to attribute “mental states” to abstract shapes interacting in short videos. We might expect these four tests to interrelate due to their shared demand on making inferences in contexts with quite explicit interpersonal interaction. However, these tests also clustered with a fifth test that involved narrative-based inferencing. In contrast to the other tests, it is less clear that narrative-based inferencing involves interpersonal interaction, as it simply requires the individual to integrate information coherently across sentences. Having said that, even in processing local-level coherence in a task such as this, we need to infer the narrator’s intention to be relevant – i.e. we expect them to maintain coherence for us in an optimal way, and not, for instance, to change setting without telling us. This suggests that if the tests interrelate because they involve making inferences about interpersonal interactions, then we should note that these interactions can be really quite implicit, such as that between a writer and an implied reader. We should be careful therefore not to identify this factor with understanding explicit interpersonal communication but rather with forming inferences more implicitly based on integrating information in context based on heuristics about how people interact.

While core language skills and inferential skills are related (in our CFA, they were correlated at 0.55), the indication that a two rather than a one factor structure underpins performance on our test battery suggests that these two sets of skills are partially dissociable in the general population. This would be expected based on the small amount of evidence that core language predicts some of the variance in pragmatic language skills both cross-sectionally ( Volden *et al.* (2009); Whyte & Nelson (2015)) and longitudinally ( Bernard & Deleau, 2007; Greenslade *et al.*, 2019; Hale & Tager-Flusberg, 2005; Miniscalco *et al*., 2014). However, these are separable domains. This agrees with the longstanding clinical intuition that some individuals (such as some of those with autism or pragmatic language impairments) can have difficulties with conversational language in the relative absence of problems with grammar and vocabulary ( Baird & Norbury, 2016). Indeed, in follow-up work, we plan to administer the same test battery to autistic adults, with the expectation that they will find the tests specifically clustered within the “social understanding” factor more difficult. This work will be useful in establishing whether our tests, especially the ICT, are useful in explaining real-life conversational difficulties. In our findings reported here, the tests generally showed low correlations, which might lead us to question whether they are really measuring general abilities that are relevant to everyday-life. However, if our tests show group differences when comparing those with and without communication challenges, then we can argue that they are sensitive to cognitive processes relevant to day-to-day communication.

Alongside this follow-up work with autistic adults, we also plan to administer a similar test battery to children, as we are interested in how pragmatic processing (as measured by the ICT) and grammar and vocabulary skills might develop in a separable or co-dependent way. Given that we enter the linguistic environment without a lexicon, it may be that we depend on a continual interaction between pragmatic and core language skills, meaning that there may be few situations in which we rely on one domain in relative exclusion of the other. Our aim in creating the ICT was to isolate as much as possible pragmatic processing by limiting the demand on core language skills; this may be possible in adults, as the linguistic code may be stored in a more self-encapsulated way as it represents “crystallized” knowledge. However, in children there may be continual interaction between core language, pragmatics, other social-cognitive skills, logical reasoning etc., to allow for the greater possibility that they might encounter unfamiliar language. This potentially differing architecture of mind may mean that pragmatic processing in the ICT is less dissociable from grammar and vocabulary skills in children. It all depends on how “modular” we take the different functions to be; certainly, pragmatic processing of communicative stimuli is seen as modular by Relevance Theory ( Carston & Powell, 2008), so with regard to the ICT, for instance, it may be that the tendency to search for relevant implicit meanings is dissociable from core language skills from an early age. This has repercussions for our understanding of developmental conditions involving a pragmatic impairment, like autism. If we assume that pragmatic and core language skills highly interact during development, then we might expect problems with pragmatics to have knock-on effects on acquisition of the linguistic code, but that there might be a piggybacking of skills in one domain on the other. This would mean that pragmatic skills would likely develop slowly but along a normal course in people with autism, especially those with well-developed core language skills. However, if pragmatic processing really is modular, then there may be more fundamental differences for those with a pragmatic impairment in how they process language involving implied meanings. There may be some compensation in these individuals which allows them to process language (as all language involves pragmatic interpretation, according to Relevance Theory), but this may involve effortful, error-prone or otherwise atypical processing. This remains speculative, and our future work may shed some light on these questions.

An unexpected finding in this study was the relatively low correlations between the language tests. Reliability of the tests was good, so this cannot be attributed to their being noisy. The only correlation showing a moderate effect size was between grammar and vocabulary, indicating that these tests hung together as measures of core language skills. All other tests were rather weakly correlated. This suggests that performance was influenced by a range of task-specific skills rather than dominated by domain-general abilities. It is particularly interesting that the Awkward Dialogues and Frith-Happé Animations were minimally correlated. Both tests would be assumed to tap advanced “theory-of-mind”/mentalizing skills. However, the lack of correlation here indicates that, at least in the general population, individual differences in performance on these tests are not accounted for by a general social cognition factor but rather by much more task-specific skills. Our findings agree with research in children and adolescents, which has found low correlations between several advanced “theory-of-mind” measures ( Hayward & Homer, 2017). This questions the coherence of “theory-of-mind” as a single construct, and it would be worthwhile exploring this issue in future research using factor analysis.

One objective of this study was to understand more about what our test of implicature comprehension measured. Core language skills, such as vocabulary and grammar, accounted for only a small proportion of variance in scores on our Implicature Comprehension Test. This means that some individuals might be able to decode the basic “literal” meaning of an utterance, as encoded by the individual words and grammatical structure, without processing an implied meaning. Such individuals would be assumed to include those with autism and related conditions, who are found to have difficulties forming inferences ( Loukusa & Moilanen (2009)); diagnostic criteria often refer to problems with non-literal/implicit meanings ( Baird & Norbury, 2016). The dissociation between implicature comprehension and core language skills is also in line with linguistic theories that describe implicature as context-dependent meaning that is not intrinsic to the linguistic code (see Grice’s theories, Relevance Theory, etc. in Ariel, 2010). We also found that an individual’s ability to make inferences, as measured in several of our tests, explained some variability in how effectively people process conversational implicature, even accounting for the role played by grammar and vocabulary skills. This suggests that there is some commonality between processing implicature in conversational interchanges and forming inferences in other contexts - and the contexts in our test battery were wide-ranging, including narratives, abstract cartoons and social dialogues.

However, the shared variance was relatively small, leaving a considerable proportion of the variability in implicature scores unexplained. What skills might explain why people varied in their scores on the test? We have no categorical answers to this, but there are a couple of things to bear in mind. First off, it is likely that interpreting implied meanings is a complex process underpinned by multiple strategies; we may use formal logic when responding to test items requiring inferences to be made, but we may also be influenced in a more automatic way by what we feel other people might choose, i.e. by social norms. As such, there may be more effortful processing involved in the latter case and also more intuitive “gut-based” responses in the latter. It should be noted, however, that items on this test were not correct simply by virtue of being selected by the most people. There was less consensus for some items on the ICT, and yet item-level accuracy tended to correlate well with test totals excluding that item. This suggests that there was some latent ability underpinning performance across items on the test. As such, if there are multiple strategies in processing implicature, including formal reasoning and sensitivity to social norms, then these strategies likely combine as a unitary process.

And what might this unitary process involve? We designed the test under the influence of Relevance Theory, and so an obvious answer might be sensitivity to the principle of communicative relevance ( Sperber & Wilson, 1986). In the context of implicature, this is the expectation that an utterance should respond relevantly to the previous contribution in a conversation, and if it doesn’t seem to, then we should be open to the possibility of the interlocutor intended us to pick up an implied meaning. It may be that some individuals are more active in seeking relevance and implied meaning, and this tendency may explain individual differences in how people detect implicature. We hope to explore this question in future research through assessing the relationships between implicature comprehension and novel tasks that involve sensitivity to the principle of relevance. One possible task might involve having participants make judgements on how relevant conversational turns are – e.g. whether the turn provides too much or insufficient information in the context of the conversation. We might expect individuals who are sensitive to utterances that are optimally relevant in their context to also be adept at picking up implied meanings suggested by the context.

In summary, this study presents evidence that understanding language in its communicative context is not simply a matter of core language skills. In particular, we found that understanding implicated meanings in conversation is somewhat distinct from vocabulary knowledge and grammatical competence. This raises the question of whether individuals with autism and social communication difficulties may have especial problems with this conversational understanding even if they perform at a typical level on tests of vocabulary and grammar. Our future work will explore this question.

## Data availability

### Underlying data

Open Science Framework: Structural and pragmatic language processing in adults. https://doi.org/10.17605/OSF.IO/XN48E ( Wilson & Bishop, 2019)

This project contains the following underlying data:

- Data
  - alldataAnalyse.R (script for collating data, and running confirmatory factor analyses and exploratory regressions)
  - AwkwardConvo.csv (data for the Awkward Dialogues)
  - Backchannel.csv (data for the Test of Fillers and Backchannels)
  - Data_dictionary.xlsx (spreadsheet detailing contents of each data file; one sheet per file)
  - Grammar.csv (data for the Grammaticality Decision Test)
  - Implicature.csv (data for the Implicature Comprehension Test)
  - Implicature_itemcodes.csv (spreadsheet detailing item-type for each item in the Implicature Comprehension Test)
  - Inferencing.csv (data for the Test of Local Textual Inference)
  - TOManimations.csv (data for the Frith-Happé Animations)
  - Vocab.csv (data for the Synonyms Test)

### Extended data

Materials for tests devised for this study are being developed as an assessment tool that we hope will be sensitive to pragmatic impairments in individuals with social communication difficulties, but we still need to establish validity of the tests in clinical groups before they are made available. We are also concerned that open availability of the materials may reduce their usefulness if participants have already viewed them prior to testing. However, we are happy to share our materials with other researchers wishing to use them. Please contact the corresponding author, with an explanation of why access is sought.

Information for researchers wishing to gain access to the Frith-Happé Animations is available here: https://sites.google.com/site/utafrith/animations. Any researchers wishing to use these animations should contact Sarah White ( s.white@ucl.ac.uk).

Open Science Framework: Structural and pragmatic language processing in adults. https://doi.org/10.17605/OSF.IO/XN48E ( Wilson & Bishop, 2019)

This project contains the following extended data:

- MaterialsGUIDE-MATERIALS.odt (this document provides a description of the files available under Materials, and provides item-level statistics for some test items.)

- Awkward Dialogues
  ○ AwkwardDialogue.mp3 (example item for the Awkward Dialogues)

- Implicature Comprehension Test
  ○ Practice_Item_1.mp4 (Example practice trial of the Implicature Comprehension test)
  ○ ICT1.mp4 (Example implicature item)
  ○ ICT2.mp4 (Example implicature item)
  ○ ICT3.mp4 (Example implicature item)
  ○ ICT4.mp4 (Example implicature item)

- Test of Fillers/Backchannels
  ○ Filler1.mp4 (Example item)
  ○ Filler2.mp4 (Example item)
  ○ Filler3.mp4 (Example item)
  ○ Filler4.mp4 (Example item)
  ○ Filler5.mp4 (Example item)
  ○ Filler6.mp4 (Example item)

- Test of Local Textual Inference
  ○ TestLocalTextualInference.docx (Full materials for the narrative inferencing test)
