# Supplementary material for: "If you catch my drift...": ability to infer implied meaning is distinct from vocabulary and grammar skills
Source: Wellcome Open Res. 2019 Aug 30;4:68. Originally published 2019 Apr 15. [Version 3] doi: 10.12688/wellcomeopenres.15210.3 (PMC6589929; doi:10.12688/wellcomeopenres.15210.3)
Supplement: Peer review history

# Peer review: Reviewer response for version 2

**DOI:** 10.21956/wellcomeopenres.16739.r35947

**Published:** 2019-07-29

**Authors:** Lauren Swineford

**Affiliations:** 1 Washington State University, Pullman, WA, USA

I appreciate the changes made in response to comments from all reviewers. No further changes are requested.

Is the work clearly and accurately presented and does it cite the current literature?

Yes

If applicable, is the statistical analysis and its interpretation appropriate?

Yes

Are all the source data underlying the results available to ensure full reproducibility?

Yes

Is the study design appropriate and is the work technically sound?

Yes

Are the conclusions drawn adequately supported by the results?

Yes

Are sufficient details of methods and analysis provided to allow replication by others?

Yes

Reviewer Expertise:

Social communication and language development.

I confirm that I have read this submission and believe that I have an appropriate level of expertise to confirm that it is of an acceptable scientific standard.

# Peer review: Reviewer response for version 2

**DOI:** 10.21956/wellcomeopenres.16739.r35946

**Published:** 2019-07-19

**Authors:** Danielle Matthews

**Affiliations:** 1 Department of Psychology, University of Sheffield, Sheffield, UK

Thanks for addressing my questions.

Is the work clearly and accurately presented and does it cite the current literature?

Yes

If applicable, is the statistical analysis and its interpretation appropriate?

I cannot comment. A qualified statistician is required.

Are all the source data underlying the results available to ensure full reproducibility?

Yes

Is the study design appropriate and is the work technically sound?

Yes

Are the conclusions drawn adequately supported by the results?

Partly

Are sufficient details of methods and analysis provided to allow replication by others?

No

Reviewer Expertise:

Pragmatic development.

I confirm that I have read this submission and believe that I have an appropriate level of expertise to confirm that it is of an acceptable scientific standard.

# Peer review: Reviewer response for version 1

**DOI:** 10.21956/wellcomeopenres.16598.r35476

**Published:** 2019-06-21

**Authors:** Katie Alcock

**Affiliations:** 1 Department of Psychology, Lancaster University, Lancaster, LA1 4YF, UK

This is an interesting and useful paper examining the development and properties of a set of tests of pragmatic language and associated skills.

 The introduction sets out the difficulty in assessing pragmatic language skills separate from other aspects of language ability, and other cognitive and social abilities, in people with autism (PWA). It also helpfully outlines the definition of pragmatics and how this definition is sometimes expanded by authors testing other social aspects of language. This topic is of interest both to those with a linguistics background and those from a cognitive framework and hence the definition of some basic terms is in order.

 The methods are clearly designed but like another reviewer, I would really like more information (perhaps in an Appendix) about some of the stimuli. I get the point that participants might perform differently if pre-exposed to the tests but sample items (that are not in the actual tests) would be helpful to follow the thread of the paper.

 Table 1 would benefit from presentation of the maximum (and minimum, where relevant) scores possible as well as the maximum achieved, to give an additional piece of information relevant to ceiling effects.

 There are some very interesting implications in the Discussion for the coherence of Theory of Mind theory. It might be interesting to expand this issue a little bit more and/or say something about its coherence in children as well.

 As the overall findings were that none of the other measures (except Filler/Backchannels) uniquely predicted variability in Implicature, it would be very helpful to know how the authors may intend to proceed to further unpack what abilities *are* related to Implicature.

 In addition, the finding that tasks did not intercorrelate but were influenced by task-specific skills leads one to wonder if they are measuring things that are generalisable to everyday life? A little more discussion on this would be welcome.

 A few minor points:

 Pg 4 paragraph 1. I think the third sentence should read ‘“I’m working all day” implicitly turns down SPEAKER TWO’s request to pick Sally up.’

 Regarding participant characteristics:

 The authors appear to be using “is/are” and “identify as” interchangeably. Participants can, in studies like this, self-declare more or less anything without proof (I am assuming no proof is asked for). The wording of questions is therefore quite important, especially since participants may decline to answer or answer differently depending on how a question is worded. In one study we asked younger parents about the wording of age-band questions in a study of infant language, for example, and younger parents did not always want to state their exact age due to feelings of being judged as a younger parent. One can envisage that a participant may *identify* as a student without in fact being one, and some individuals find it impossible or uncomfortable to “identify as” male or female as their sex is a matter of fact not identity. It is therefore helpful to know the precise source/wording of these questions (and also, for future research, to consider whether the wording used will get accurate and complete answers).

Is the work clearly and accurately presented and does it cite the current literature?

Yes

If applicable, is the statistical analysis and its interpretation appropriate?

Yes

Are all the source data underlying the results available to ensure full reproducibility?

Yes

Is the study design appropriate and is the work technically sound?

Yes

Are the conclusions drawn adequately supported by the results?

Yes

Are sufficient details of methods and analysis provided to allow replication by others?

Partly

Reviewer Expertise:

Developmental psychology, language development, vocabulary development.

I confirm that I have read this submission and believe that I have an appropriate level of expertise to confirm that it is of an acceptable scientific standard.

# Response

**Published:** 2019-07-11

**Authors:** Alexander Wilson

**Affiliations:** University of Oxford, UK

Thank you very much for your review. We respond below to any comments that have not been addressed in our responses to the other reviewers.

- “Table 1 would benefit from presentation of the maximum (and minimum, where relevant) scores possible as well as the maximum achieved, to give an additional piece of information relevant to ceiling effects.”

We assume you mean Table 4, which presents descriptive statistics for the main study. We have now included this information.

- “There are some very interesting implications in the Discussion for the coherence of Theory of Mind theory. It might be interesting to expand this issue a little bit more and/or say something about its coherence in children as well.”

We agree that there are implications for theory of mind. It should be noted, however, that the Awkward Dialogues was not designed specifically to measure theory of mind, so we need to be tentative here. Our discussion already refers to a study finding low correlations between different theory of mind measures in children. We have added a suggestion that future work could more systematically investigate the factor structure of theory of mind tasks.

- “As the overall findings were that none of the other measures (except Filler/Backchannels) uniquely predicted variability in Implicature, it would be very helpful to know how the authors may intend to proceed to further unpack what abilities *are* related to Implicature.”

We have no categorical answers to this question, but elaborate on this issue at the end of our Discussion.

- “In addition, the finding that tasks did not intercorrelate but were influenced by task-specific skills leads one to wonder if they are measuring things that are generalisable to everyday life? A little more discussion on this would be welcome.”

This is a fair criticism, and we appreciate that there are questions of ecological validity in relation to our tests. We would argue that the issue of the tests’ usefulness hinges on whether there are group differences between autistic and non-autistic groups in our follow-up work. If there are, that is a strong argument for the relevance of our tests.

- “Pg 4 paragraph 1. I think the third sentence should read ‘“I’m working all day” implicitly turns down SPEAKER TWO’s request to pick Sally up.’”

Thanks for pointing out the error in this sentence. We have amended SPEAKER TWO to SPEAKER ONE.

- Regarding participant characteristics: “The authors appear to be using “is/are” and “identify as” interchangeably. Participants can, in studies like this, self-declare more or less anything without proof (I am assuming no proof is asked for). The wording of questions is therefore quite important, especially since participants may decline to answer or answer differently depending on how a question is worded. In one study we asked younger parents about the wording of age-band questions in a study of infant language, for example, and younger parents did not always want to state their exact age due to feelings of being judged as a younger parent. One can envisage that a participant may *identify* as a student without in fact being one, and some individuals find it impossible or uncomfortable to “identify as” male or female as their sex is a matter of fact not identity. It is therefore helpful to know the precise source/wording of these questions (and also, for future research, to consider whether the wording used will get accurate and complete answers).”

Our demographic information was collected in response to open questions asking participants what their age, gender and race/ethnicity were. We hoped that this format would give participants the opportunity to identify in ways that felt most appropriate to them. We chose to avoid the term “identify” in the questions themselves, as this is likely to mean different things to different people – indeed, as you suggest. Thank you for pointing out that we use “are/is” and “identify as” interchangeably in describing our participants; this was motivated by a view that people are how they identify. However, for consistency, in our report, we have amended all uses of “is/are” to language around “identifying as”/“reporting”, as this is the nature of the information we have. Regarding student status, this information is derived from a question asking participants whether they were currently studying. The response options were: “yes (at high school/secondary school)”, “yes (at university as an undergraduate)”, “yes (at university as a postgraduate)”, “yes (undertaking vocational training)” and “no”. The motivation behind this question was to see what proportion of our sample was students – due to the student bias in psychology samples.

# Peer review: Reviewer response for version 1

**DOI:** 10.21956/wellcomeopenres.16598.r35325

**Published:** 2019-05-23

**Authors:** Lauren Swineford

**Affiliations:** 1 Washington State University, Pullman, WA, USA

This study examined the independence of pragmatic skills from core language skills in a sample of 120 adults. This study used newly developed language tasks including the Implicature Comprehension Test designed to measure comprehension of conversational implicature. This paper was well written, and the amount of detail provided across sections was much appreciated.

 The introduction to this study provides a thorough overview of the theoretical background relevant to designing an assessment to solely measure pragmatic processing and provides sufficient rationale for why assessment of comprehension of conversational implicature could be used as a measure of pragmatic abilities that could be separable from core language abilities. The authors follow the theoretical discussion with a brief introduction into their study including clear hypotheses.

 The information provided in the methods section is detailed and describes all important aspects for the reader; this paper is a great model of a complete methods section with the inclusion of statistical code. The proposed statistical plan is appropriate for the data and the research hypothesis. One concern I had in regard to the methods was related to the validity of some tasks to measure other aspects of language. For example, assessments such as the Synonyms Test or the Grammaticality Decision Test were noted to be devised for this study. The description of these assessments provides adequate detail about these tasks/assessments, but when examining the independence/relationship between different aspects of language (core vs. pragmatic), more well-known, validated assessments would strengthen the analyses and results. I would not expect the authors to recollect data using different assessments at this point as I do not see this as a fatal flaw, especially since they examine and report the item level functioning and reliability of measures, but perhaps including a discussion of why these newer assessments were used over currently available (with strong psychometrics) tools. Also, more information would be useful on what the authors believe the other assessments set to load onto the pragmatics factor measure as the goal was to test how well the ICT measures solely pragmatics rather than broader social communication (mental state, TOM, etc) which seem to be measured by other assessments included. Perhaps providing all item level information of the assessments in a supplement, rather than just a few examples in the methods, would help to interpret the factor loadings and correlations. The detail (including whether or not assumptions for statistical analyses were met) was appropriate. Standing alone, it is not clear from Figure 2 what the different shades of color/types of lines are.

 I appreciate the authors discussion of their findings, especially regarding what the pragmatic factor in their final two-factor model may measure given the loading of narrative-based inferencing. The authors do a nice job of discussing the results in light of the theoretical discussion laid out in the introduction. I would love to hear more discussion about the next steps based on the findings in the discussion.

Is the work clearly and accurately presented and does it cite the current literature?

Yes

If applicable, is the statistical analysis and its interpretation appropriate?

Yes

Are all the source data underlying the results available to ensure full reproducibility?

Yes

Is the study design appropriate and is the work technically sound?

Yes

Are the conclusions drawn adequately supported by the results?

Yes

Are sufficient details of methods and analysis provided to allow replication by others?

Yes

Reviewer Expertise:

Social communication and language development.

I confirm that I have read this submission and believe that I have an appropriate level of expertise to confirm that it is of an acceptable scientific standard, however I have significant reservations, as outlined above.

# Response

**Published:** 2019-07-11

**Authors:** Alexander Wilson

**Affiliations:** University of Oxford, UK

Thank you for your comments. Below we address any suggestions not captured in our response to the first reviewer.

- “One concern I had in regard to the methods was related to the validity of some tasks to measure other aspects of language. For example, assessments such as the Synonyms Test or the Grammaticality Decision Test were noted to be devised for this study. The description of these assessments provides adequate detail about these tasks/assessments, but when examining the independence/relationship between different aspects of language (core vs. pragmatic), more well-known, validated assessments would strengthen the analyses and results. I would not expect the authors to recollect data using different assessments at this point as I do not see this as a fatal flaw, especially since they examine and report the item level functioning and reliability of measures, but perhaps including a discussion of why these newer assessments were used over currently available (with strong psychometrics) tools.”

As we were collecting data online, it was not possible to use existing measures due to copyright issues in presenting them online. It should also be noted that such measures would typically have been validated for face-to-face administration on a one-to-one basis, and so we cannot assume that they would have identical properties if given online. We have mentioned these issues in our Data Analysis section.

- “Also, more information would be useful on what the authors believe the other assessments set to load onto the pragmatics factor measure as the goal was to test how well the ICT measures solely pragmatics rather than broader social communication (mental state, TOM, etc) which seem to be measured by other assessments included. Perhaps providing all item level information of the assessments in a supplement, rather than just a few examples in the methods, would help to interpret the factor loadings and correlations.”

Issues pertaining to this comment are more thoroughly discussed in our response to the first reviewer under points 3 and 4. However, to briefly respond to this comment, we have clarified what skills we intended to measure, and how these reflect our model of language processing, in the Introduction, and we provide more examples of items at different levels of difficulty in Extended Data. As noted in our paper, we have ongoing work, so we prefer not to make our tests fully available in case participants see them prior to testing. However, we hope that showing more examples will help the reader conceptualise what the tests are measuring. Please note that Table 5 presents some useful item-level statistics.

- “I would love to hear more discussion about the next steps based on the findings in the discussion.”

We briefly discuss related work with autistic adults and with children in our Discussion.

# Peer review: Reviewer response for version 1

**DOI:** 10.21956/wellcomeopenres.16598.r35322

**Published:** 2019-04-29

**Authors:** Danielle Matthews

**Affiliations:** 1 Department of Psychology, University of Sheffield, Sheffield, UK

I enjoyed reading this paper. Pre-registration and access to the data and R script was a plus. With the exception of the request for more details about the stimuli/items (point 4 – the most important point for me), the following comments are mainly points for discussion (that could be accommodated with clarification in the discussion section or with minor revision to the introduction) not suggestions for substantial revision. I’ve commented on the paper with a focus on the logic of the arguments put forward. While I have looked at the R script and the data, I haven’t run the script myself. I haven’t commented on the stats beyond a couple of questions (about the relation between test development and factor analysis, and about how to interpret the factor analysis).

 The goal of this study was “to devise novel language tests to give a more convincing answer to the question of whether core language abilities and sensitivity to social aspects of language are separable sets of skills in the general adult population.” A definition of pragmatics is given and the need to contrast this with semantics discussed. “While semantics involves decoding conventional “dictionary” meaning, pragmatics is all about inference: we use context to infer further non- codified meaning.” While there are theoretical issues here (relating to what ‘conventional dictionary meaning’ is), this is a working definition. Particularised conversational implicatures are settled on as the ideal domain for testing pragmatics given their dependence on communicative context. The goal was to use a p.c. implicature test to find out how far pragmatic skill is separable from other language abilities. 

1. It is argued that a focus on p.c. implicatures would be beneficial since conceptually it is the most distinctively pragmatic skill (whereas other skills that are nominally pragmatic have more potential overlap with, e.g., semantics). Does this mean that the results (finding a 2 factor solution) are not necessarily what would be found if we looked at other skills traditionally considered as pragmatic? I wondered to what extent picking the most distinctively pragmatic skill (the one at the far end of the continuum, so to speak) makes it difficult to assess the main question of whether core language abilities are separable from pragmatic abilities (in general). 
2. Likewise, does use of CTT and IRT (where during test development items are excluded for poor fit) bias things in favour of finding separable factors? Essentially, the steps here are to devise a test, e.g., with a set of items that theoretically at least all require p.c.implicature. Then any items that do not pattern with most of the others are left out. This is desirable for test development. But I did wonder whether doing this at the same time as factor analysis essentially means that you have chosen items on the basis that they pattern together and then shown in factor analysis that they do. I doubt given the number of items excluded that this is really a problem but it might be worth clarification (whether this is an issue in principle or not, whether it is an issue in practice or not).  
3. Given the goal of testing whether formal language skills group separately to pragmatic language skills, to what extent are the four ‘conversation comprehension’ tests cleanly tests of pragmatics? I can see the implicature test is a test of pragmatics (at least as far as it is possible to have a clean test, there is a convincing rationale here). But why are the Frith-Happé Animations – a test of mental state attribution intended to be as non-verbal as possible - taken as a measure of conversation comprehension? The discussion recognised this as a somewhat separate test. It seemed odd to go to great lengths to attempt to identify a cleanly pragmatic test (to the exclusion of tests of other nominally pragmatic skills) and then to include a test traditionally seen as tapping ToM and not involving conversation comprehension at all.  
  Likewise, for the Awkward Dialogues, p.6 reads  “The test was designed not so much to measure a single skill but rather to tap general conversational competence, including pragmatics, mental state attribution, and understanding of paralinguistic cues such as intonation, as well as core language skills.” Could you clarify why this would be helpful for addressing the question at hand?
  It seems ‘pragmatics’ – already a broad tent - is being stretched further than necessary here. To find out whether that is the case perhaps exploratory factor analysis would help but a broader range of tests would be needed. For this paper, perhaps some further rationale could be given along with a revision to the introduction. (I had followed the introduction – largely focused on the implicature test – and then was surprised when I came to Table 3 at the choice of conversation comprehension measures).
4. Provision of test items. It was great to be able to read this paper, look at the pre-reg document, data and R code. What would have made it really great, and what is perhaps more important for conceptualising the findings, would have been able to see the full list of items from the tests. Without this, I found myself struggling to imagine what was going on exactly. The example p.c. implicatures given seem fairly straightforward such that I guess most of the adults who participated answered correctly. However, there was variance for this measure, so some of the inferences must have been harder to make. I’d have been interested to get a sense of these items, not least because it is a challenge to make a difficult inference test item that has a correct answer. It is reported that some items on this test were more difficult than others and that scores for a given difficult item correlated well with total scores excluding that item, indicating that they reliably tapped a particular skill. I’d like to better understand what the skill is exactly. I guess it could be distracting to give one example that a reader could take issue with for reasons that are beside the point, but given the problems particular to this sub-field, being able to concretely point to what items had in common seems important. What would be really valuable would be to see the full set of stimuli with the difficult items highlighted so that we could try to imagine what those items have in common. 
**Suggested revision: **Ideally, the full stimuli sets/list of items would be given so that the we can understand the tasks. It is noted in a final section of the paper (‘Extended data’) that stimuli are not provided as test development is underway and future participants might access materials. I would be interested to see the full list but, for publication, would it be possible to give a few example items from the harder end of each test, with information about what % of participants got them ‘right’? This is needed for the implicature, awkwardness, fillers and narrative tests. 
  More generally – perhaps for discussion - does it always make sense to say there is a correct answer? Or just one that most people alight on? For example, with the filler/give the floor test, there are probabilistic cues to who will speak next. Is the correct answer just the answer that most people would give? Are we looking at norms of behaviour (a participant is correct by virtue of doing what most other people would do) or is there a clear inferential process that should optimally be engaged in? 
5. P.9 “Any individual with at least one outlying score on any test was excluded from the dataset". How many were excluded? Could this be noted in the participants section also?
6. In the results section, it wasn’t entirely clear what question the exploratory analysis was intended to address. Could this be spelt out somewhere?   
7. **Conclusions drawn.**  I was interested in the discussion on p. 12 about what the second factor (non-grammar factor) represents. I wondered if it might be possible to interpret the CFA a little for people who don’t use it regularly. Does this analysis show there is a second factor that holds together or is it just that a two factor solution is better than having just one factor, one sensible factor is grammar (with vocab) and another dimension is the inferencing test (with other tests also grouping with this but not really strongly related to the second factor). That is, this seems to show that p.c. implicature and grammar are quite distinct. But does it tell us that p.c. implicature and the other tests that are nominally tapping “pragmatic/communication” should be conceptualised as a single factor ‘inferring meaning through integrating information in context’? Looking at the correlation matrix and the loadings, these variables seem quite loosely related. In answer to the main question of “whether core language abilities and sensitivity to social aspects of language are separable sets of skills in the general adult population”, it seems the answer is yes but apparently only in so far as grammar is distinct from p.c. inferencing (which is reflected in the title and final conclusions). What everything else is doing, and what elements of the other nominally conversation measures are pragmatic (or social aspects of language use) seemed less clear to me. Some additional text explaining what this factor analysis allows us to conclude here (and how future studies could further test whether there is a pragmatics factor and if so what it reflects) would be very helpful.   
  In sum, the title and main conclusion about inferencing and grammar are supported but the question set up in the introduction had a broader scope and the conclusion wrt to that broader question could be clarified. 
8. I was interested in the implications of this study for development. Would you predict separation of vocabulary/grammar and pragmatics earlier in development? Given that children start off without a ‘dictionary/code/lexicon’, perhaps some consideration of the co-development of these skills would be helpful for understanding developmental disorders.
9. Typo: well-correlated twith  >> with

Is the work clearly and accurately presented and does it cite the current literature?

Yes

If applicable, is the statistical analysis and its interpretation appropriate?

I cannot comment. A qualified statistician is required.

Are all the source data underlying the results available to ensure full reproducibility?

Yes

Is the study design appropriate and is the work technically sound?

Yes

Are the conclusions drawn adequately supported by the results?

Partly

Are sufficient details of methods and analysis provided to allow replication by others?

No

Reviewer Expertise:

Pragmatic development.

I confirm that I have read this submission and believe that I have an appropriate level of expertise to confirm that it is of an acceptable scientific standard, however I have significant reservations, as outlined above.

# Response

**Published:** 2019-07-11

**Authors:** Alexander Wilson

**Affiliations:** University of Oxford, UK

Many thanks for your comments on the paper. Please see below for our responses.

- Regarding the focus on p.c. implicatures rather than other pragmatic skills: “I wondered to what extent picking the most distinctively pragmatic skill (the one at the far end of the continuum, so to speak) makes it difficult to assess the main question of whether core language abilities are separable from pragmatic abilities (in general).” 

This is an interesting point, and I think comes down to the issue of what we mean by pragmatic skills. Our argument is that pragmatic comprehension involves context-dependent inference (as per Relevance Theory), and so testing the question of whether pragmatics is distinct from core language requires a “pure” measure of context-dependent inference. We observe that many existing tests of pragmatics conflate pragmatics with other domains, so testing the relationship between performance on these tests and core language skills will ultimately only tell us that tests involving a significant demand on core language skills interrelate. It is also interesting that you use the metaphor of a continuum for pragmatics (vs. semantics, I assume). I think the language model that we are testing would look more like a Venn diagram, with overlapping domains for core language and pragmatic processing. Most tests would tap the central overlapping section, but we are trying as much as possible to design a test that puts most of its cognitive demand on the pragmatic domain. We have included more detail in our Introduction about our model of language processing (which is ultimately heavily influenced by Relevance Theory).

- "Does use of CTT and IRT (where during test development items are excluded for poor fit) bias things in favour of finding separable factors?"

The purpose of using reliability analysis was to minimise the amount of measurement error in each test. This would mean that scores on our tests of implicature and grammar, for instance, represented as far as possible participants’ “true scores” in these domains and were less subject to random error caused by poor items that did not reflect the domain well (perhaps because they did not have a “right” answer or were ambiguous in some way). If we’re looking to test the relationship between, say, implicature and grammar, it makes sense to ensure that the tests measure these constructs with as little error as possible. Indeed, if implicature and grammar are closely related, then we would expect that removing random error would increase, not decrease, correlations between domains, as error attenuates true correlations. As such, we’re not sure that conducting reliability analysis leads to the bias described.

- "Given the goal of testing whether formal language skills group separately to pragmatic language skills, to what extent are the four ‘conversation comprehension’ tests cleanly tests of pragmatics?"

The tests (besides the implicature comprehension test) were included not so much as “clean” tests of pragmatics, but instead to establish convergent validity between the implicature test and other tests that we might expect to tap pragmatics (as well as other skills), based on the idea that pragmatics involves sensitivity to communication contexts and is thought to be a subdomain of “theory-of-mind” under Relevance Theory. We include some further clarification on this in our Introduction. Please note that in our pilot study we found that the implicature test was unrelated to anything, so we wanted to establish whether it did relate to other tests with a pragmatic demand, as a means of exploring the nature of the construct. We have also renamed this factor as “social understanding”, as we feel that more appropriately reflects the tests, and also is more in line with the theoretical basis.

- "Ideally, the full stimuli sets/list of items would be given so that the we can understand the tasks. It is noted in a final section of the paper (‘Extended data’) that stimuli are not provided as test development is underway and future participants might access materials. I would be interested to see the full list but, for publication, would it be possible to give a few example items from the harder end of each test, with information about what % of participants got them ‘right’? This is needed for the implicature, awkwardness, fillers and narrative tests." 

In writing up this paper, we were conscious of needing to restrict access to our stimuli as we have ongoing work, and wanted to reduce the possibility of participants seeing our tests in advance of testing. You’re right, however, that we have not shared our materials sufficiently for the results to be fully interpreted. We have taken your advise and included as extended data five implicature items with item-level statistics. All five of the awkward dialogues were of approximately the same difficulty; we share one dialogue in extended data with the associated questions. We share a few videos for the fillers task, with item-level statistics. The inferencing test is less important to our follow-up work. The whole test is now available as extended data.

- "More generally – perhaps for discussion - does it always make sense to say there is a correct answer? Or just one that most people alight on?"

This is a valid point. To some extent we are looking at consensus answers with some of the tests. With the fillers task, this is particularly true – although, in many ways the consensus answer can also be seen as the “correct answer”, as individuals who alight on the non-consensus answer are likely to find themselves disadvantaged during communication with individuals who are sensitive to normative cues. We have provided some speculation in the Discussion about the role of norms and formal reasoning in our implicature test.

- "How many [participants] were excluded? Could this be noted in the participants section also?"

We have included all excluded scores in our results table, and note the number of participants included in our analysis in Results. For completeness, we now include the number of excluded participants in the participants section too.

- "In the results section, it wasn’t entirely clear what question the exploratory analysis was intended to address. Could this be spelt out somewhere?"

The exploratory analysis was intended to quantify the amount of overlap between the implicature test and the other tests (especially when accounting for core language skills). We felt this was important given that our pilot study had shown no relationships between the implicature test and other measures, so we wanted to establish the extent to which it was tapping a general ability that might be reflected in other tests of communication/social understanding. Essentially, we were asking the question as to how specific/general the test was. We have clarified this in the Results.

- In relation to the Discussion: “Does this analysis […] tell us that p.c. implicature and the other tests that are nominally tapping “pragmatic/communication” should be conceptualised as a single factor ‘inferring meaning through integrating information in context’?  Looking at the correlation matrix and the loadings, these variables seem quite loosely related. [...] Some additional text explaining what this factor analysis allows us to conclude here (and how future studies could further test whether there is a pragmatics factor and if so what it reflects) would be very helpful. […] In sum, the title and main conclusion about inferencing and grammar are supported but the question set up in the introduction had a broader scope and the conclusion to that broader question could be clarified."

We think it is fair to question the extent to which the tests grouped under the communication factor really represent one general ability in this domain. As you point out, the correlations between the tests were relatively low, indicating that task-specific skills were important in determining performance on individual tasks, with a more general inferential ability accounting for a small amount of the variance in scores. We have clarified this in the conclusion, and have more distinctly addressed the questions of (a) implicature dissociating from grammar/vocab and (b) core language dissociating from pragmatics.

- "I was interested in the implications of this study for development. Would you predict separation of vocabulary/grammar and pragmatics earlier in development? Given that children start off without a ‘dictionary/code/lexicon’, perhaps some consideration of the co-development of these skills would be helpful for understanding developmental disorders."

This is an interesting point, and is relevant to some of our follow-up work. We provide some exploration of this in our Discussion.

- "Typo: well-correlated twith >> with".

Corrected.
